# Quantitative Measurement of GPCR Endocytosis via Pulse-Chase Covalent Labeling

**DOI:** 10.1371/journal.pone.0129394

**Published:** 2015-05-28

**Authors:** Hidetoshi Kumagai, Yuichi Ikeda, Yoshihiro Motozawa, Mitsuhiro Fujishiro, Tomohisa Okamura, Keishi Fujio, Hiroaki Okazaki, Seitaro Nomura, Norifumi Takeda, Mutsuo Harada, Haruhiro Toko, Eiki Takimoto, Hiroshi Akazawa, Hiroyuki Morita, Jun-ichi Suzuki, Tsutomu Yamazaki, Kazuhiko Yamamoto, Issei Komuro, Masashi Yanagisawa

**Affiliations:** 1 Department of Cardiovascular Medicine, Graduate School of Medicine, The University of Tokyo, Tokyo, Japan; 2 Department of Molecular Genetics, Howard Hughes Medical Institute, University of Texas Southwestern Medical Center, Dallas, Texas, United States of America; 3 Department of Advanced Clinical Science and Therapeutics, The University of Tokyo, Tokyo, Japan; 4 Department of Gastroenterology, Graduate Scholl of Medicine, The University of Tokyo, Tokyo, Japan; 5 Department of Allergy and Rheumatology, Graduate School of Medicine, The University of Tokyo, Tokyo, Japan; 6 Max Plank- The University of Tokyo Center for Integrative Inflammology, The University of Tokyo, Tokyo, Japan; 7 Department of Diabetes and Metabolic Diseases, Graduate School of Medicine, The University of Tokyo, Tokyo, Japan; 8 Clinical Research Support Center, The University of Tokyo, Tokyo, Japan; 9 International Institute for Integrative Sleep Medicine (WPI-IIIS), University of Tsukuba, Tsukuba, Japan; University of York, UNITED KINGDOM

## Abstract

G protein-coupled receptors (GPCRs) play a critical role in many physiological systems and represent one of the largest families of signal-transducing receptors. The number of GPCRs at the cell surface regulates cellular responsiveness to their cognate ligands, and the number of GPCRs, in turn, is dynamically controlled by receptor endocytosis. Recent studies have demonstrated that GPCR endocytosis, in addition to affecting receptor desensitization and resensitization, contributes to acute G protein-mediated signaling. Thus, endocytic GPCR behavior has a significant impact on various aspects of physiology. In this study, we developed a novel GPCR internalization assay to facilitate characterization of endocytic GPCR behavior. We genetically engineered chimeric GPCRs by fusing HaloTag (a catalytically inactive derivative of a bacterial hydrolase) to the N-terminal end of the receptor (HT-GPCR). HaloTag has the ability to form a stable covalent bond with synthetic HaloTag ligands that contain fluorophores or a high-affinity handle (such as biotin) and the HaloTag reactive linker. We selectively labeled HT-GPCRs at the cell surface with a HaloTag PEG ligand, and this pulse-chase covalent labeling allowed us to directly monitor the relative number of internalized GPCRs after agonist stimulation. Because the endocytic activities of GPCR ligands are not necessarily correlated with their agonistic activities, applying this novel methodology to orphan GPCRs, or even to already characterized GPCRs, will increase the likelihood of identifying currently unknown ligands that have been missed by conventional pharmacological assays.

## Introduction

G protein-coupled receptors (GPCRs) contain the characteristic seven-transmembrane domain and represent one of the largest families of signal-transducing receptors [1]. GPCRs play a critical role in many physiological systems by responding to various ligands such as nucleotides, amines, lipids and peptides. The number and functional activity of GPCRs at the cell surface influences the cellular responsiveness to GPCR ligands, and the number of GPCRs available for activation, in turn, is dynamically controlled by receptor trafficking [2].

Agonist-induced conformational changes trigger the activation of canonical G protein-mediated signaling and phosphorylation of the activated receptors [3]. The interaction of phosphorylated GPCRs with ß-arrestins followed by receptor accumulation in clathrin-coated pits (CCPs) is the critical event that initiates agonist-induced GPCR internalization. ß-arrestins are scaffolding molecules recruited from the cytosol to the activated receptors at the cell surface [4]. ß-arrestins not only inhibit the interaction of GPCRs with G proteins for signal desensitization, but also promote GPCR internalization by binding to the activated receptors and to structural components of the CCPs.

The endocytic trafficking pathway of individual GPCRs is remarkably varied. Some GPCRs appear to traffic efficiently to lysosomes after ligand-induced endocytosis. These receptors exhibit attenuated responsiveness to their cognate ligands after prolonged and/or repeated ligand stimulation because a significant fraction of the overall receptor pool is degraded by the lysosomes. In contrast, some GPCRs have been shown to be stable after prolonged and/or repeated ligand stimulation, suggesting that they immediately recycle back to the plasma membrane after endocytosis. This second type of GPCR has the ability to recover quickly from a period of desensitization and thereby maintains cellular responsiveness to the cognate ligands. Thus, distinct endocytic trafficking routes of otherwise similar GPCRs lead to different functional effects on cellular responsiveness to GPCR ligands.

Although physiological ligands of GPCRs are generally considered to promote their cognate receptor endocytosis, some drugs that stimulate the same GPCRs do not efficiently trigger receptor endocytosis even when applied at saturating concentrations. For example, mu opioid receptors are known to undergo rapid endocytosis following stimulation by endogenous opioid neuropeptides, but some non-peptide agonists, such as morphine, do not elicit receptor endocytosis [5–7]. Notably, recent studies have shown that the endocytic activities of GPCR ligands do not necessarily correlate with their relative agonistic activities as measured with conventional pharmacological analyses.

As described above, GPCR endocytosis critically affects GPCR signaling, and the endocytic trafficking patterns are, in turn, linked specifically to individual receptors and/or ligands. Therefore, it is critical to develop experimental techniques that enable us to measure GPCR internalization on a routine basis. Antibodies against receptors or high-affinity ligands tagged with fluorophores have been utilized successfully for this purpose [8]. However, it remains unclear if antibodies or ligands are continuously bound to GPCRs once they have been internalized into a cell. To overcome this limitation and further improve the simplicity, sensitivity and flexibility of the assay, we used HaloTag technology [9,10].

**Fig 1:**
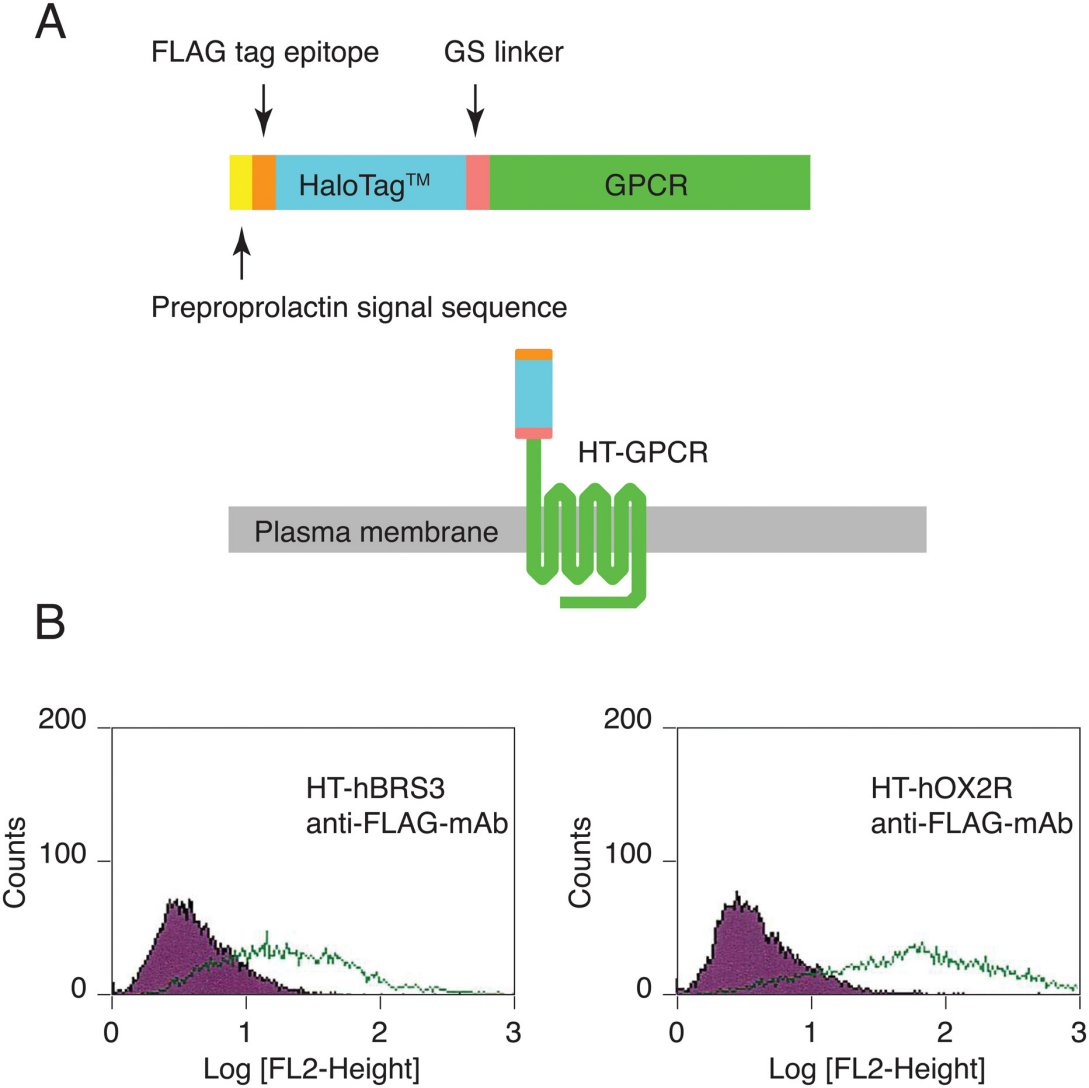
HT-GPCRs and their cell surface expression. (A) A schematic of HT-GPCR. (B) FACS analyses of CHO cells stably expressing HT-hBRS3 and HT-hOX2R. Cells were stained with an anti-FLAG mAb followed by incubation with PE-labeled anti-mouse IgG antibodies. The open histograms represent CHO-HT-GPCR cells and the filled histograms represent CHO-mock cells.

The HaloTag is a catalytically inactive derivative of a bacterial hydrolase. This artificial tag has the ability to form an irreversible covalent bond with synthetic HaloTag ligands, which contain fluorophores or a high-affinity handle (such as biotin) in addition to a HaloTag reactive linker. We genetically engineered a chimeric GPCR by fusing the HaloTag to the N-terminal end of the receptor (HT-GPCR) (Fig 1A). Utilizing a HaloTag PEG ligand that does not cross the cell membrane efficiently because of its hydrophilic spacer with four ethylene glycols, we were able to selectively pulse-chase label HT-GPCRs expressed on the cell surface at any given moment. This non-radioactive covalent labeling of GPCRs allowed us to develop a novel assay to directly measure the relative number of GPCRs internalized into cells.

## Materials and Methods

### Plasmids and cells

HT-GPCR chimeras consisting of the bovine prolactin signal sequence (MDSKGSSQKGSRLLLLLVVSNLLLCQGVVS), followed by a FLAG epitope tag (DYKDDDDK), the HaloTag sequence without the first methionine, the GS linker (G_4_S x 3) and the GPCR sequence without the first methionine (Fig 1A) were subcloned into a pMXs-IRES-Neo retroviral vector [11]. These plasmids were then individually transfected into the retrovirus packaging cell line Plat-E [12]. The supernatant from the transfected cells was removed 48–72 h later and applied to CHO cells expressing the ecotropic receptor (CHO-ER). Transduced cells were selected and maintained in the presence of the appropriate antibiotics (G418 sulfate, Invitrogen) (1mg/ml). CHO cells were grown in DMEM supplemented with 5% FCS and 1% nonessential amino acids (Invitrogen).

### Pulse-chase covalent labeling of HT-GPCRs on the cell surface

CHO cells stably expressing the HT-GPCR of interest were plated in 96-well plates (2–4 x 10^4^ cells/well). After 24 h, 5 μM of the HaloTag PEG-Biotin Ligand (Promega) was added to the wells and incubated for 15 min at 37°C. After incubation, cells were washed three times in fresh medium (DMEM supplemented with 5% FCS and 1% nonessential amino acids) to eliminate unreacted HaloTag ligands.

### Flow cytometry analysis

CHO cells stably expressing HT-GPCRs were treated with HaloTag PEG-Biotin Ligand (final conc. 10 μM) (Promega) for 15 min at 37°C. Cells were washed three times with PBS and then incubated with streptavidin phycoerythrin (SA-PE, BD Biosciences) for 20 min on ice. Ten thousand cells per sample were analyzed using a FACS Calibur instrument (BD Biosciences).

### Quantitative measurement of HT-GPCRs on the cell surface

After pulse-chase labeling of HT-GPCRs, GPCR ligands were added to the wells and incubated for 1 h. Following incubation, cells were washed three times in PBS and fixed in 4% paraformaldehyde (PFA) for 30 min at room temperature (RT). After washing in PBS, fixed cells were incubated with streptavidin horseradish peroxidase (SA-HRP, Pierce) (0.2 μg/ml) for 20 min at RT. After removal of unreacted SA-HRP by washing three times in PBS, cells were incubated with HRP substrates (1-Step Ultra TMB-ELISA, Pierce) for 20 min at RT. The reaction was stopped by the addition of 2 N H_2_SO_4_ before measuring the optical density (OD) at 450 nm with a plate reader (Victor V, Perkin Elmer), according to the manufacturer’s instructions.

### Quantitative measurement of internalized HT-GPCRs

After pulse-chase labeling of HT-GPCRs, GPCR ligands were added to the wells and incubated for 1 h. Following incubation, cells were washed three times in PBS and fixed in 4% paraformaldehyde (PFA) for 30 min at RT. After washing, fixed cells were incubated with SA (Pierce) (5 μg/ml) for 20 min at RT. Cells were then incubated with 0.3% Triton X-100 for 10 min at RT to permeabilize the cell membrane. To eliminate internal peroxidase activities in the permeabilized cells, cells were treated with 0.3% H_2_O_2_/PBS for 30 min at RT. Cells were then incubated with SA-HRP (0.2 μg/ml) for 20 min at RT. After removal of unreacted SA-HRP by washing three times in PBS, cells were incubated with HRP substrates for 20 min at RT. The reaction was stopped by adding 2 N H_2_SO_4_ before measuring the optical density (OD) at 450 nm with a plate reader (Victor V, Perkin Elmer).

### Statistical Analyses

Results are expressed as the mean ± SEM. Data involving multiple groups were assessed by ANOVA with Dunnett’s multiple comparison of means test.

## Results

### Successful expression of HT-GPCRs on the cell surface

GPCRs genetically fused to the HaloTag, HT-GPCRs, were constructed and subcloned into a pMXs-IRES-Neo vector (Fig 1A). To facilitate their cell surface expression, the preproprolactin signal sequence was inserted after the first ATG codon of the HT-GPCRs. Cell lines stably expressing individual HT-GPCRs were established through retroviral infection of CHO-ER cells. To test whether chimeric HT-GPCRs were expressed on the cell surface, cells were examined by flow cytometry after staining with an anti-FLAG mAb. We studied two different HT-GPCRs: HT-human bombesin receptor subtype 3 (HT-hBRS3) and HT-human orexin type 2 receptor (HT-hOX2R). As expected, both chimeric receptors were successfully expressed on the cell surface (Fig 1B).

### Pulse-chase covalent labeling of HT-GPCRs expressed on the cell surface

We next performed pulse-chase labeling of HT-GPCRs utilizing the HaloTag PEG-Biotin Ligand. This ligand with a hydrophilic spacer does not cross the cell membrane efficiently, and therefore is able to form an irreversible covalent bond selectively with the HT-GPCRs expressed on the cell surface. Because it remains unclear whether endosome-localized GPCRs are continuously bound to antibodies or ligands, covalent labeling via the HaloTag technology is advantageous. Stable covalent labeling not only allows us to perform extensive washing to reduce background, but also ensures that the labeled GPCRs can be tracked until they become degraded in the lysosome.

**Fig 2:**
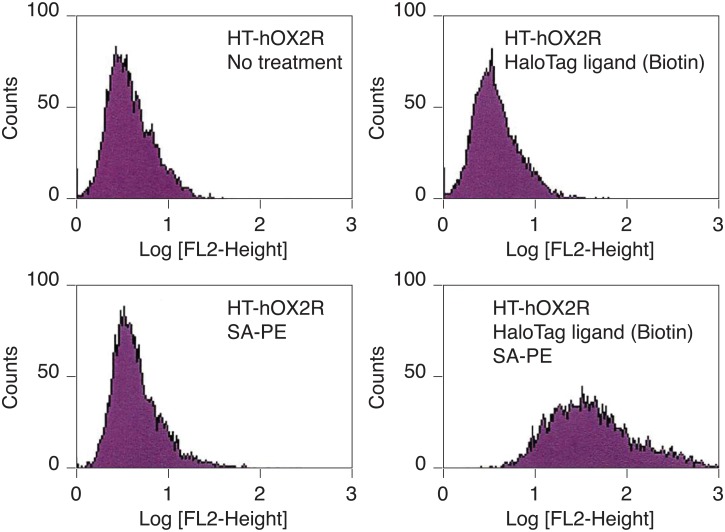
Pulse-chase covalent labeling of HT-GPCRs expressed on the cell surface. Cells expressing HT-hOX2R were labeled with the HaloTag PEG-Biotin Ligand.

Following incubation of cells expressing chimeric HT-GPCRs with the HaloTag PEG-Biotin Ligand, cells were incubated with streptavidin phycoerythrin (SA-PE, BD Biosciences) and analyzed by flow cytometry. Because we had already confirmed the expression of HT-hOX2R on the cell surface (Fig 1B), we studied HT-hOX2R to test whether the HaloTag ligand is able to label HT-GPCRs. As expected, the HaloTag PEG-Biotin Ligand successfully labeled HT-hOX2R (Fig 2). These results suggest that HT-GPCRs on the cell surface can be pulse-chase labeled at any given moment by utilizing a HaloTag PEG ligand.

### Measurements of GPCR turnover rates

**Fig 3:**
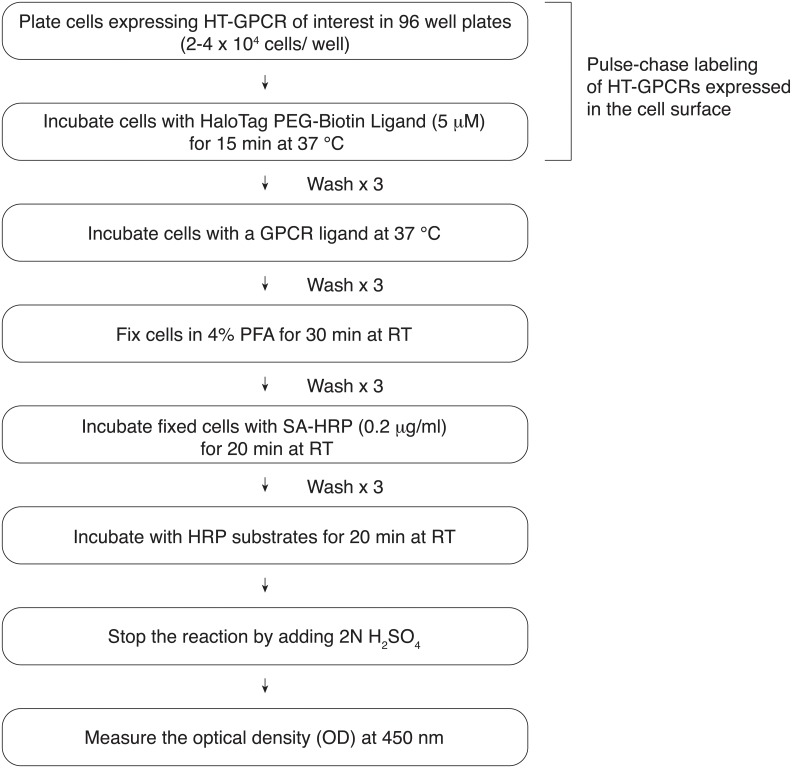
A brief overview of the protocol for monitoring the relative number of GPCRs at the cell surface.

**Fig 4:**
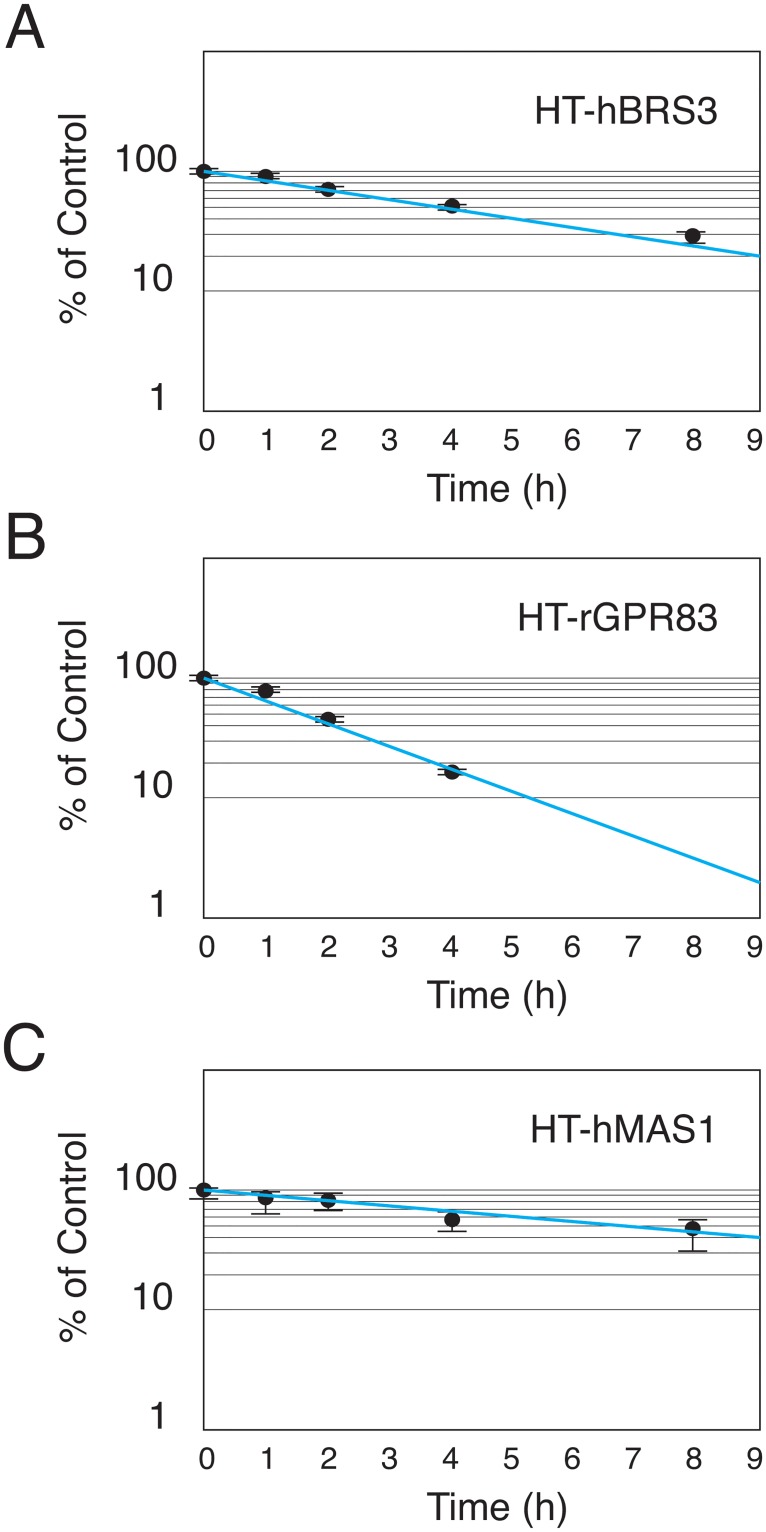
GPCR turnover rates. Turnover rates of individual GPCRs are shown. (A) HT-hBRS3, (B) HT-rGPR83 and (C) HT-hMAS1. Percentage of internalized receptor was calculated as the OD values at 450 nm of each point divided by those of a negative control (0 h incubation). Representative data (mean ± SEM) from at least 3 independent experiments performed in triplicate are shown. ** p<0.01, one-way ANOVA with Dunnett’s multiple comparison test.

We then utilized the above techniques for pulse-chase covalent labeling of HT-GPCRs to study GPCR turnover rates. Three GPCRs, hBRS3, rat GPR83 (rGPR83) and human MAS1 (hMAS1), were randomly selected for our experiments. Cells stably expressing individual HT-GPCRs were pulse-chase labeled with the HaloTag PEG-Biotin Ligand and fixed in 4% PFA at different time points (0, 1, 2, 4 and 8 h). Fixed cells were then incubated with SA-HRP. After adding the HRP substrates, the optical density (OD) was measured at 450 nm to quantitatively monitor the relative number of receptors remaining at the cell surface (Fig 3). The elimination half-life from the cell surface for hBRS3, rGPR83 and hMAS1 was 4 h, 1.7 h and 8 h, respectively (Fig 4). Consistent with the previous notion that the endocytic trafficking patterns of individual GPCRs are remarkably different [2], these results suggested that each GPCR has its own basal turnover rate from the cell surface even in the absence of its cognate ligands.

### Monitoring agonist-induced GPCR internalization

**Fig 5:**
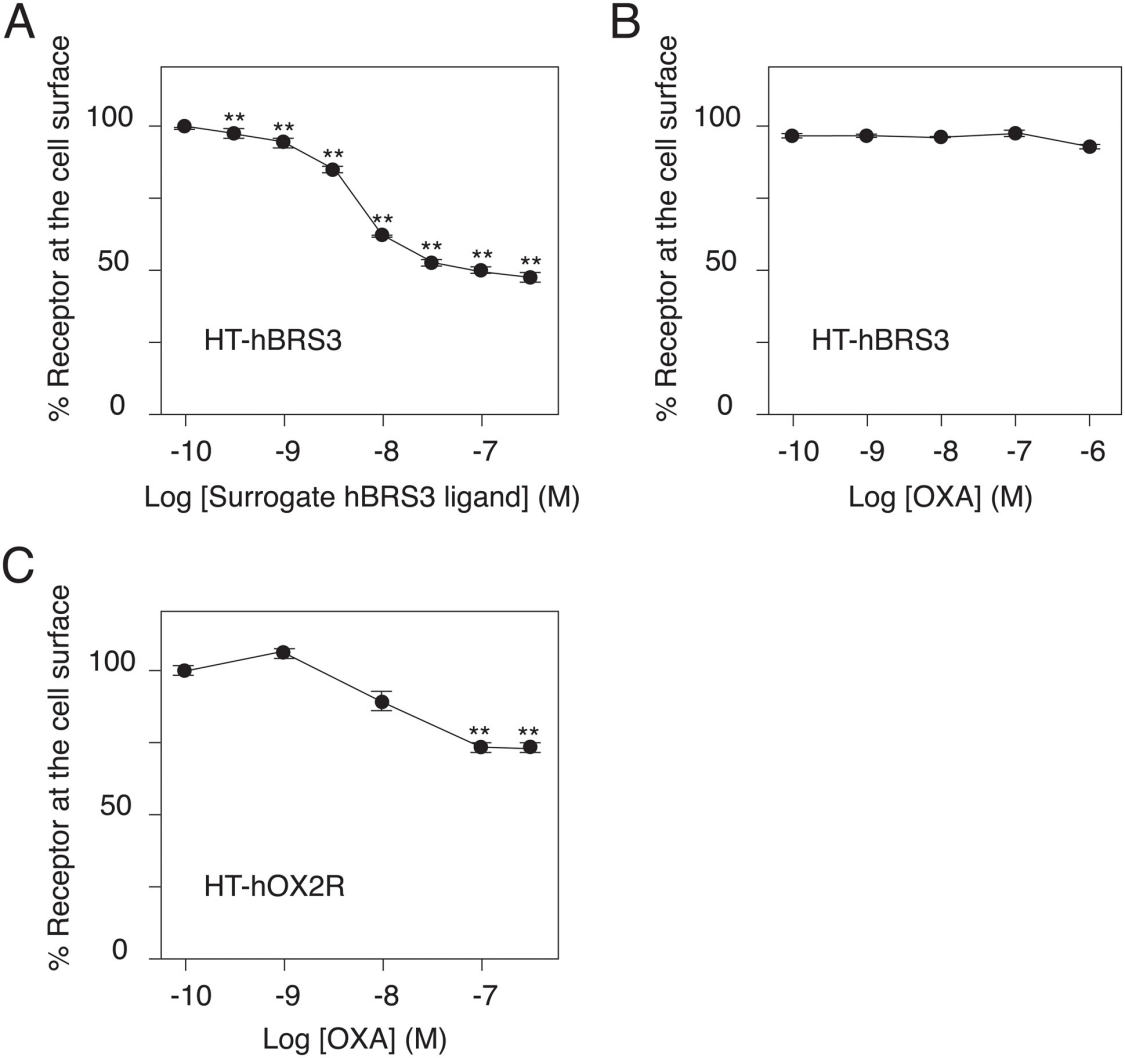
Agonist-induced GPCR internalization. One hour of agonist stimulation decreased the number of GPCRs at the cell surface in a dose-dependent manner (A and C), whereas irrelevant ligand stimulation did not change the number of GPCRs at the cell surface (B). HT-hBRS3 (A and B) and HT-hOX2R (C). Percentage of labeled receptor at the cell surface was calculated as the OD values at 450 nm of each point divided by those of a negative control (no ligand stimulation). Representative data (mean ± SEM) from at least 3 independent experiments performed in triplicate are shown. ** p<0.01, one-way ANOVA with Dunnett’s multiple comparison test.

GPCR turnover rates exhibit considerable diversity among receptors, and some GPCRs, including rGPR83, have less than a 2 h elimination half-life (Fig 4). We therefore studied agonist-induced GPCR internalization by stimulating the receptors for an hour. After pulse-chase labeling of HT-GPCRs on the cell surface with the HaloTag PEG-Biotin Ligand, cells were incubated with various concentrations of agonists. Following one hour of agonist stimulation, cells were immediately fixed and stained with SA-HRP to measure the number of receptors remaining on the cell surface through the HRP enzymatic reaction. HT-hBRS3 and HT-hOX2R were stimulated by surrogate hBRS3 ligands ([D-Phe^6^, ß-Ala^11^, Phe^13^, Nle^14^]-Bombesin (6–14); Phoenix Pharmaceuticals, Inc.) and orexin A peptides (OXA, American Peptide Company), respectively. Agonist stimulation decreased the number of the labeled receptors on the cell surface in a dose-dependent manner, suggesting that both agonists triggered their respective cognate receptor internalization (Fig 5 and S1 Fig).

**Fig 6:**
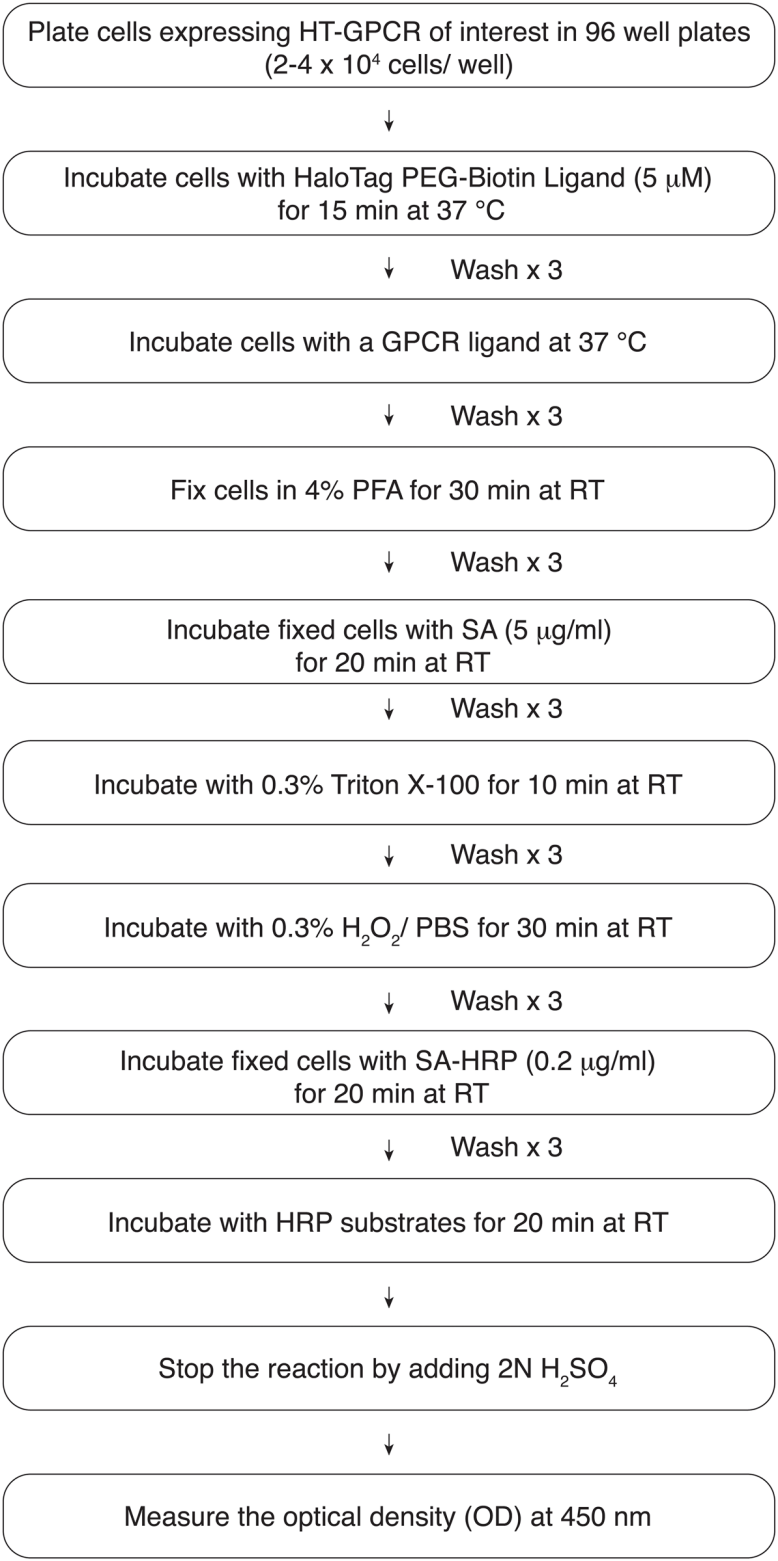
A brief overview of the protocol for directly monitoring the relative number of GPCRs internalized into cells.

**Fig 7:**
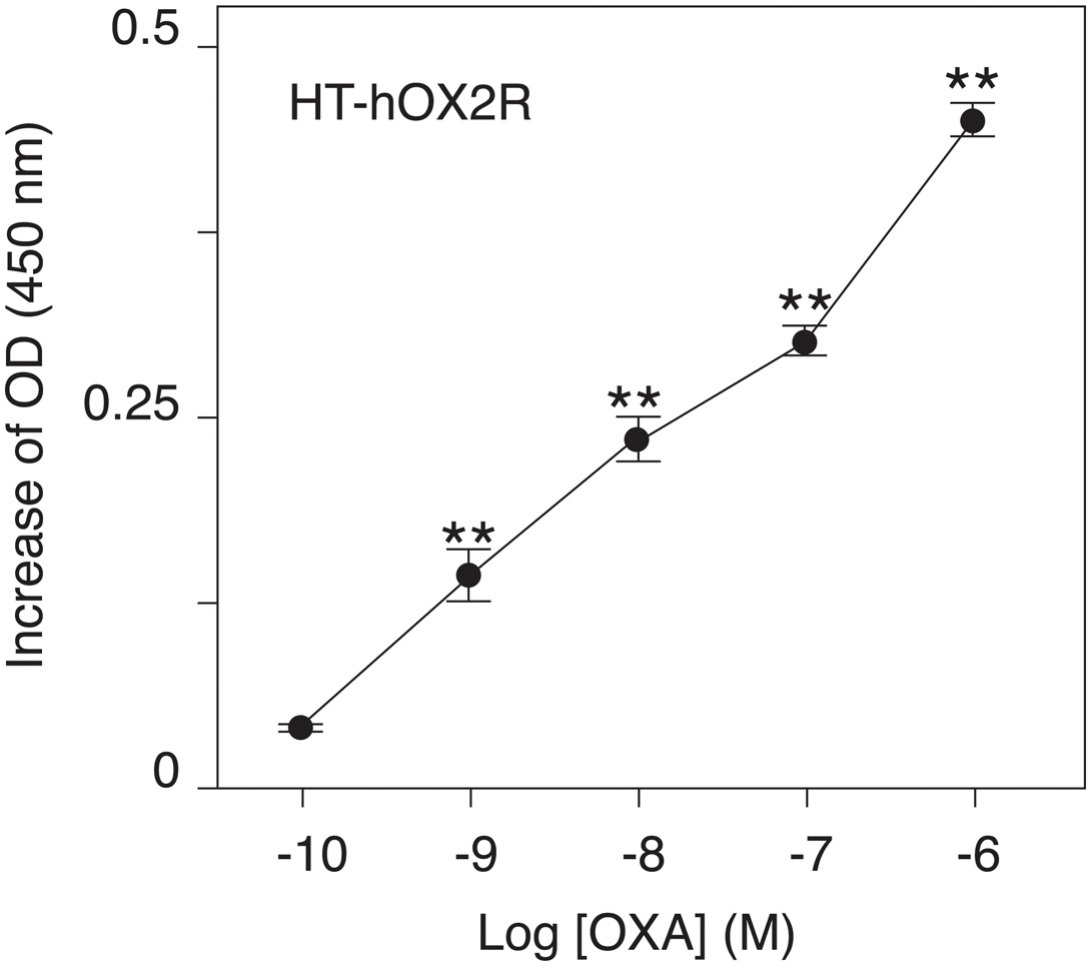
Direct measurement of the relative number of internalized GPCRs. Cells were stimulated with various concentrations of OXA for 1 h. OXA stimulation increased the number of HT-hOX2Rs internalized into the cell in a dose-dependent manner. Representative data (mean ± SEM) from at least 3 independent experiments performed in triplicate are shown. ** p<0.01, one-way ANOVA with Dunnett’s multiple comparison test.

Agonist stimulation decreased the number of HT-hOX2Rs on the cell surface to a smaller extent than the number of HT-hBRS3s. To improve the signal-to-noise (S/N) ratio of the HT-hOX2R internalization assay, we therefore established a modified protocol for direct measurement of the relative number of the internalized receptors (Fig 6). After agonist stimulation, cells were immediately incubated with SA to block all the free biotin sites remaining on the cell surface. Cell surface membranes were then permeabilized to make the internalized HT-hOX2Rs, which had been labeled with the HaloTag PEG-Biotin Ligand, accessible to the SA-HRP. We observed that OXA stimulation increased the number of internalized HT-hOX2Rs in a dose dependent manner, suggesting that this modified protocol enables us to directly measure the relative number of internalized receptors (Fig 7). In our original assay, the number of HT-hOX2Rs on the cell surface was significantly decreased when cells were stimulated with 100 nM of OXA (Fig 5C). Interestingly, however, we could detect a significant increase in agonist-induced receptor internalization even when cells were stimulated with 1 nM of OXA with this modified protocol (Fig 7). When compared with our original methodology, this revised protocol was thus more sensitive in detecting receptor internalization.

## Discussion

In the present study, we developed a novel GPCR internalization assay utilizing HaloTag technology. This assay enabled us to pulse-chase label GPCRs on the cell surface at any given moment, and track the labeled receptor after endocytosis.

Previous studies have demonstrated that endocytic elimination of GPCRs from the cell surface leads to attenuated cellular responsiveness to their cognate ligands in the acute phase, but promotes later functional recovery from the desensitized state through receptor recycling. Interestingly, recent studies have indicated that endosome-localized GPCRs can also activate G proteins and that internalized GPCRs support a significant component of G protein-mediated signaling in the acute phase (i.e., for several minutes after agonist stimulation) [13,14]. These findings have revised a long-held belief that GPCRs are able to interact with G proteins only at the plasma membrane.

Given the contribution of GPCR endocytosis to acute GPCR signaling and its classical roles in receptor desensitization and resensitization, studying the endocytic behavior of individual GPCRs as induced by various ligands is more important than ever. Our newly developed assay utilizes HaloTag technology to improve the sensitivity, specificity and versatility compared to classical GPCR internalization assays, and therefore will facilitate the pharmacological characterization of GPCR endocytosis. In general, this assay can be applied to any GPCR. Importantly, however, the effects of attaching the HaloTag protein to the N-terminus of receptors must be carefully evaluated especially for some GPCRs that recognize their ligands through the N-terminal sequence.

Considering that endocytic activities of GPCR ligands are not necessarily correlated with their pharmacological agonistic activities, applying this methodology to orphan GPCRs, or even to already characterized GPCRs, will increase the likelihood of identifying currently unknown ligands that have been missed by conventional G protein assays. The discovery of small compounds that do not activate canonical G protein signaling but modulate receptor endocytosis has significant therapeutic potential. For example, enhancing the ability of mu opioid receptors to recycle to the cell surface by administration of a small compound with such characteristics may improve pathophysiological tolerance to the antinociceptive effects of opioids [15].

An important caveat to our study is that functional data obtained by the characterization of GPCR endocytic behavior *in vitro* may not be relevant to physiological endocytosis occurring *in vivo*. To overcome this limitation, it would be important to create a mouse model harboring a HT-GPCR “knocked-in” into the genomic locus of the corresponding receptor. Previous work has demonstrated that knock-in mice expressing fluorescent GPCR (GPCR-EGFP) provide a useful approach for studying receptor trafficking through dynamic visualization of the receptor [16]. However, receptor labeling cannot be controlled temporally in this model, because the fluorophore is genetically attached to the receptor. In contrast, HT-GPCRs can be labeled at any given moment by injecting HaloTag ligands into HT-GPCR knock-in mice, which would allow us to perform *in vivo* experiments involving time-resolved pulse-chase analyses. By utilizing this knock-in mouse model combined with the HaloTag ligands, it would be possible to examine how *in vivo* GPCR trafficking is influenced by drugs and/or physiological stimuli that trigger the release of endogenous ligands, and how alterations in GPCR endocytic behavior *in vivo*, in turn, affect physiological parameters.

## Supporting Information

S1 Fig Reduced cell surface expression of HT-hBRS3 after surrogate hBRS3 ligand stimulation (1 μM) was validated by FACS analysis. After pulse-chase labeling of HT-hBRS3 at the cell surface with the HaloTag PEG-Biotin Ligand, cells were stimulated with surrogate hBRS3 ligands ([D-Phe^6^, ß-Ala^11^, Phe^13^, Nle^14^]-Bombesin (6–14); Phoenix Pharmaceuticals, Inc.). Following 3 h stimulation, fixed cells were stained with SA-APC (BioLegend) and analyzed by FACSVantage SE (BD Biosciences). Data are representative of two independent experiments. The red open histogram, no agonist stimulation; the blue open histogram, agonist stimulation (1 μM); the filled histogram, negative control. The horizontal bar indicates the FACS gate. Percentages of cells within the FACS gate are shown for each group.
(EPS)
